# Climate change, gut microbiome, and epilepsy—New paradigms beyond the gut-brain axis

**DOI:** 10.3389/fneur.2025.1726561

**Published:** 2026-01-23

**Authors:** Andrea Santangelo, Antonio Corsello, Gianmichele Villano, Maria Cristina Diana, Pasquale Striano

**Affiliations:** 1 Department of Neurosciences Rehabilitation, Ophthalmology, Genetics, Maternal and Child Health (DINOGMI), University of Genoa, full member of ERN EpiCARE, Genoa, Italy; 2 Pediatric Neurology and Muscular Diseases Unit, IRCCS Istituto “Giannina Gaslini”, Genoa, Italy; 3 Department of Clinical-Surgical, Diagnostic and Pediatric Sciences, University of Pavia, Pavia, Italy; 4 Department of Clinical Sciences and Community Health, University of Milan, Milan, Italy

**Keywords:** climate change, epilepsy, gut-brain axis, microbiota, pediatric neurology, climate-gut -brain axis

## 1 Introduction

The gut–brain axis plays a key role in many neurological disorders (1), including epilepsy, Alzheimer's disease (2, 3), Parkinson's disease (4, 5), and multiple sclerosis (6). Notably, dysbiosis, an imbalance in gut microbiota, has been associated with differences in seizure susceptibility, severity and treatment response (7, 8). Microorganisms, which constitute most of the Earth's biodiversity and underpin essential biogeochemical cycles, are highly responsive to climatic variability. Changes in temperature, precipitation patterns, and humidity, particularly when characterized by extreme intensity, prolonged duration, or rapid deviations from local climatic norms as well as heatwaves defined relative to regional climatology and extreme weather events such as storms, floods, or droughts, have been shown to alter microbiome composition, diversity, and metabolic function across terrestrial, aquatic, and host-associated ecosystems. For example, rising temperatures and altered seasonal patterns have been shown to influence gut microbial communities by modifying food quality, pathogen exposure, and host metabolic and immune physiology, with downstream impacts on health in both humans and animals. Climate-induced fluctuations in environmental microbiomes also affect the reservoir and stability of microorganisms available to colonize the host, while extreme climatic stress can select for thermotolerant or rapidly adaptable taxa, contributing to community restructuring.

Despite accumulating evidence that climate variables correlate with microbial alterations, current understanding remains fragmented and largely descriptive. Crucially, there is a pronounced lack of integrative mechanistic models that unify environmental drivers, microbiome dynamics, neuroinflammation, and neurological outcomes. This gap limits our ability to predict how ongoing climatic shifts may influence microbiome-mediated processes relevant to epilepsy.

The primary conceptual thread of this topic, linking climate change, microbial dysbiosis, neuroinflammatory pathways, and seizure susceptibility, has not been clearly stated in the existing literature and remains insufficiently articulated across studies. A schematic conceptual model illustrating how climatic factors influence microbiota composition, how these microbial changes modulate neuroinflammation, and how this cascade ultimately affects seizure susceptibility would substantially strengthen the interpretative framework of this field. Such a model is essential to clarify assumptions, identify mechanistic gaps, and guide future experimental and clinical investigations.

Although climate change affects human health through multiple pathways, its potential role in modulating gut microbial ecosystems relevant for epilepsy has not been systematically explored.

We advance an integrative hypothesis that climate-related exposures may influence epilepsy vulnerability by reshaping the gut microbiome and its downstream gut–immune–brain signaling. Specifically, we propose an hypothetical causal chain in which climate stressors such as heat extremes, humidity variability, and climate-linked environmental pressures promote dysbiosis and altered microbial metabolism [including short-chain fatty acids (SCFA) depletion], leading to increased gut permeability, inflammatory cytokine signaling (IL-6, IL-1β, and TNF-α), oxidative stress, and neurotransmitter imbalance, thereby lowering seizure threshold and exacerbating seizure outcomes.

## 2 Gut-brain axis and epilepsy: is it just nutrition?

The gut-brain axis involves complex interactions between the gastrointestinal tract and the central nervous system. Studies have shown significant differences in gut microbiome composition between epileptic patients and healthy controls, with alterations in bacterial phyla such as *Bacteroidetes, Actinobacteria, Firmicutes, Proteobacteria*, and *Fusobacteria* (9, 10). Interestingly, this dysbiosis is reversible, reducing or disappearing in cases with good seizure control (8, 11).

In fact, the microbiome composition is sensitive to dietary interventions, which can be particularly effective in treating epilepsy, such as the ketogenic diet, which can induce a 50% reduction in seizures in patients with Dravet syndrome, Doose syndrome, GLUT-1 deficiency, or Infantile Spasms (12). Clinical and preclinical studies have documented various modifications in the microbiome following the introduction of this diet, such as an increase in *Bacteroidetes* and *Akkermansia* in patients who experienced clinical benefit with no significant impact on the nutritional status (13, 14). Such changes can be achieved due to the ability of gut microbiota to influence neuroinflammation and neuronal hyperexcitability, potentially modulating seizure activity. For instance, the ketogenic diet, known for its efficacy in treating drug-resistant epilepsy, alters gut microbiota composition, suggesting a therapeutic avenue through microbiome modulation (15, 16). Additionally, probiotic interventions have shown promise in reducing seizure frequency, further supporting the gut-brain connection (17).

Nonetheless, preclinical studies have highlighted how the saprophytic intestinal population can regulate serum levels of various neurotransmitters (11, 13, 18), contributing to the excitatory homeostasis of the CNS through its modulatory effect on cytokine concentrations, serotonin precursors, and SCFA (8). Recent multi-omics work demonstrates that microbiota-driven neuroinflammation involves IL-6, TNF-α, and IL-1β activation, impaired antioxidant systems, altered glutamate/GABA turnover, and SCFA depletion. In fact, pollutant-induced dysbiosis models show elevation of IL-6, oxidative stress markers, glial activation, and behavioral hyperexcitability mediated by gut-brain axis disruption (19). These mechanisms overlap with established epileptogenic pathways, strengthening the biological plausibility of a climate-sensitive microbiota–neuroinflammation–seizure axis. Several authors have identified the gut-brain axis as a potential therapeutic target for epilepsy patients. The microbiome is indeed sensitive to various environmental factors, allowing for multi-level interventions to determine its composition (20). Temperature-sensitive microbial responses are now recognized as a major determinant of host physiological stability. Microbiota composition shifts predictably across thermal gradients, influencing both host thermal tolerance and vulnerability to stress (21). As shown in ectothermic models, microbiome alterations under heat stress reduce resilience and amplify inflammatory and oxidative pathways; mechanisms that may analogously compromise seizure thresholds in susceptible patients. Clinical studies have also demonstrated the benefits of certain therapeutic strategies, such as fecal microbiota transplantation or the use of probiotics in epilepsy patients (22–24).

### 2.1 Climate change and microbiome: potential mechanisms and future directions

Climate change, characterized by rising temperatures, altered precipitation patterns, and increased frequency of extreme weather events, poses significant threats to human and animal health. Recent evidence suggests that environmental factors, including temperature and humidity, can influence gut microbiome composition both in human and in animals (25, 26). Shifts in variability, precipitation, and seasonality can affect digestion, thermal tolerance, and disease susceptibility, while also providing potential adaptive benefits (25). For example, seasonal variations and exposure to ultraviolet light have been shown to affect microbial diversity (27, 28).

Temperature fluctuations can alter the *Firmicutes* to *Bacteroidetes* ratio, a critical indicator of gut health (29). Given the gut microbiota's sensitivity to environmental changes, it is plausible that climate change could exacerbate dysbiosis, potentially impacting epilepsy management. For instance, hyperthermia has been associated with increased seizure susceptibility, potentially mediated by changes in gut microbiota and immune responses (26, 30).

The scientific community indicates that climate change, particularly global warming, poses a tangible threat to our ecosystem and health. The impact of these changes already affects various aspects of clinical activity and the therapeutic management of patients with non-communicable diseases. Among these, epilepsy represents a major research topic, affecting globally 50 million patients, with a pediatric prevalence ranging from 3.2 to 44 cases per 1,000 inhabitants, 75% of whom are from developing countries (31, 32).

Understanding the impact of climate change on this population is therefore of relevant importance to the scientific community. It is not surprising that numerous efforts have already been made in this direction. To date, some authors have theorized the impact of global warming on the therapeutic management of epilepsy patients, highlighting potential issues in drug storage, which in some cases require specific temperatures and humidity, as well as potential problems that could affect the distribution chain (33–35). An emerging concept relevant to this field is that the microbiome can exhibit strong within-habitat stability despite environmental warming, as shown by long-term multi-omics studies in permafrost peatlands (36). This suggests that only beyond specific disturbance thresholds (e.g., extreme heatwaves, dehydration, exposure to pollutants) microbiome communities may undergo state shifts.

Pre-clinical studies have shown that hyperthermia itself can predispose to the development of seizures through various mechanisms, such as altering ion channel permeability (30), activating the innate immune system, with the increased epileptogenic activity of IL-1β and TNF (37), or through hyperventilation-induced alkalosis, strictly related with climate change and global warming (38). Moreover, various syndromes frequently encountered by epileptologists have a strong association with exposure to even slightly elevated temperatures, such as fever, temperature fluctuations, immersion in hot water, or physical exercise. These conditions often have a genetic etiology associated with mutations in temperature-sensitive ion channels, such as the SCN1A gene, clinically correlated with Dravet syndrome, as well as SCN1B, GABRG2, GABRD, or CHD2 (39–41).

Several authors have also highlighted how climate change can directly or indirectly favor exposure to epileptogenic triggers. Global warming appears to lead to possible sleep reduction, increased fatigue, and increased stress related to poorer quality of life or catastrophic events (42–44). These conditions have already been observed during the current SARS-CoV-2 pandemic and are among the main factors precipitating epileptic seizures (45, 46).

Nonetheless, oxidative stress represents an additional critical pathway linking climate-induced dysbiosis to neuronal hyperexcitability. Experimental evidence shows that alterations in gut microbial composition can significantly modulate ROS accumulation, mitochondrial dysfunction, and downstream inflammatory cascades. For instance, in a zebrafish Alzheimer model, hydrogen-rich water reduced oxidative stress, neuroinflammation, and dysbiosis, revealing a tight interdependence between microbial balance and redox homeostasis (47). These findings support the hypothesis that climate-related stressors likeheat, UV exposure, dehydration, may worsen seizure susceptibility via an oxidative-stress-driven disruption of the gut–brain axis.

Additionally, it should be added that climate change may increase the incidence of febrile seizures as neurological complications of vector-borne infections, such as malaria, neurocysticercosis, or arbovirus infections (41). Current literature clearly defines that the effects of climate change on epilepsy can manifest in multiple aspects: a predisposition to seizure development through the increase of potential triggers, increasing risk factors in predisposed patients, or modifying the epidemiology of certain diseases.

**Figure 1:**
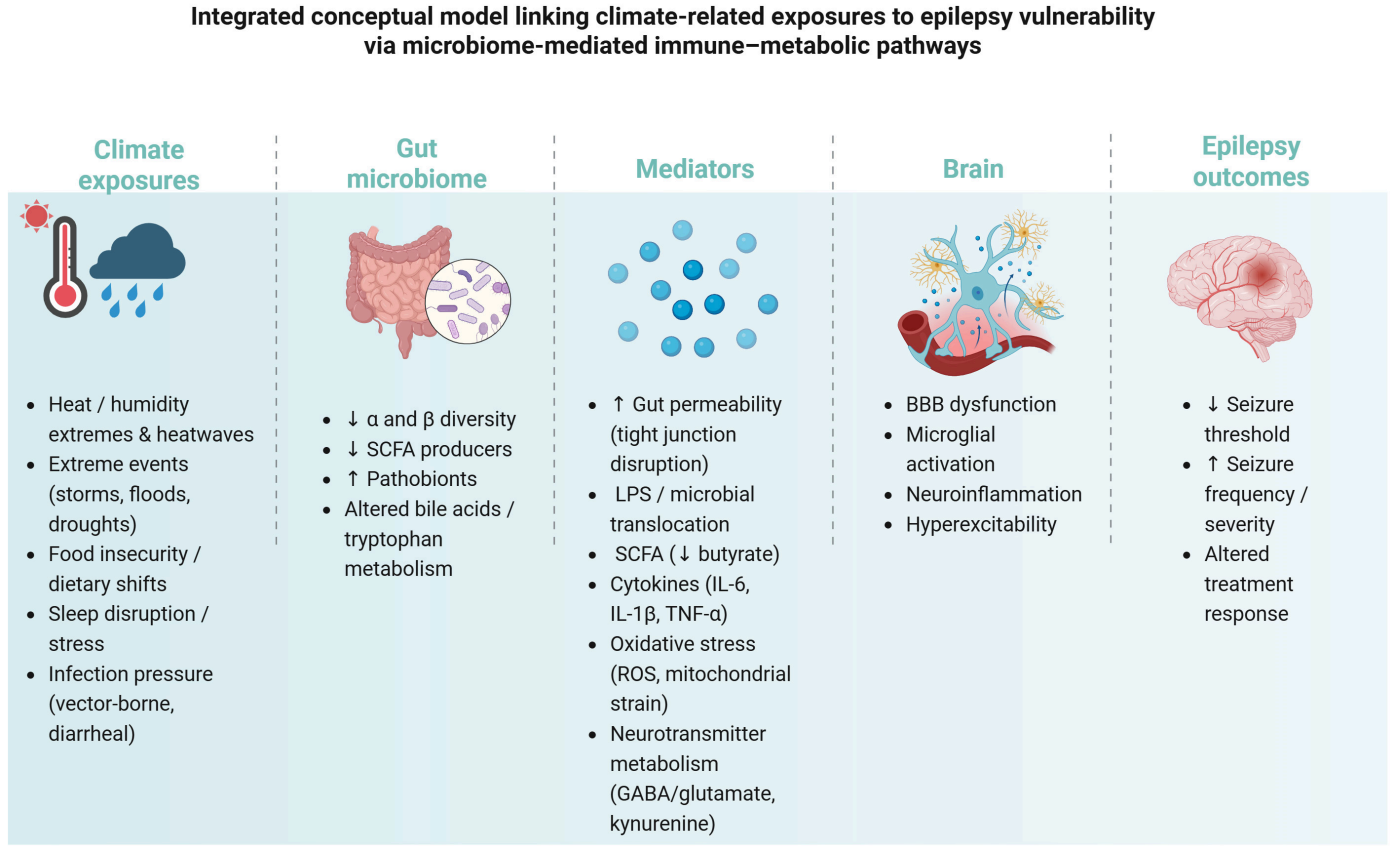
The Climate–Gut–Brain Axis. Climate stressors may drive dysbiosis (reduced diversity, loss of Short Chain Fatty Acids (SCFA) producers, expansion of pathobionts) and altered microbial metabolism, influencing gut permeability and microbial translocation (LPS), inflammatory cytokines (IL-6, IL-1β, TNF-α), oxidative stress, and neurotransmitter pathways. These mediators converge on Blood Brain Barrier (BBB) dysfunction, microglial activation, neuroinflammation, and network hyperexcitability, reducing seizure threshold and worsening seizure outcomes.

The interplay between climate change, gut microbiome, and epilepsy may involve several mechanisms (Figure 1). Elevated temperatures could directly impact gut microbiota diversity, promoting dysbiosis and influencing seizure thresholds. Climate-induced stressors, such as reduced sleep quality and increased fatigue, may further exacerbate seizure activity through neuroinflammatory pathways (42, 43, 48).

Finally, climate-related stressors, can also impair complementary pathways such as the glymphatic system, responsible for clearing metabolic waste from the brain (49). Recent work by Buguet and colleagues highlights the link between extreme heat, disrupted sleep, and glymphatic function, emphasizing the need for strategies that protect brain health in vulnerable groups (50). Therefore, measures such as proper hydration, temperature regulation, and careful medication management may be crucial for epileptic patients with impaired glymphatic clearance that can further worsen their condition. Recent controlled-environment studies demonstrate that environmental toxicants and temperature fluctuations can induce dysbiosis, oxidative stress, glial activation, and neurotransmitter imbalance, all reversible by microbiome-targeted interventions (19). These findings provide a mechanistic substrate for how climate-associated stressors may modulate seizure risk via microbial and neuroimmune pathways.

Future research should focus on longitudinal studies to assess climate change's long-term effects on the gut-brain axis in epilepsy. Investigating the impact of specific environmental factors on gut microbiota and seizure outcomes will be crucial. Additionally, exploring therapeutic interventions, such as probiotics and dietary modifications, tailored to mitigate climate change's adverse effects on the gut microbiome, holds promise for improving epilepsy management. For instance, microbiome-targeted interventions may become relevant in climate-sensitive epilepsy phenotypes. Principles from environmental microbiome engineering suggest that modifying microbial community structure through ecological or therapeutic inoculation can confer system-level resilience (51). Translating such ecological strategies to clinical settings could inspire novel probiotic or postbiotic therapies aimed at stabilizing the MGBA under climatic stress.

## 3 Discussion

The potential influence of climate change on the gut-brain axis and epilepsy represents an emerging area of research that warrants further investigation. Building on the integrative hypothesis outlined above, we propose that climate-related exposures may influence epilepsy vulnerability by reshaping the gut microbiome and its downstream gut–immune–brain signaling. Specifically, this framework predicts a causal chain in which climate stressors such as heat extremes, humidity variability, and climate-linked environmental pressures may promote dysbiosis and altered microbial metabolism, leading to increased gut permeability, inflammatory cytokine signaling, oxidative stress, and neurotransmitter imbalance, thereby lowering seizure threshold and exacerbating seizure outcomes. Understanding these interactions can provide valuable insights into developing novel therapeutic strategies and public health policies. Addressing climate change's impact on health, particularly in vulnerable populations such as those with epilepsy, requiring a multidisciplinary approach integrating environmental science, microbiology, neurology, and public health.

Microorganisms form the ecological backbone of life on Earth and respond rapidly to environmental perturbations. Alterations in temperature, humidity, and precipitation can modify microbial composition, richness, and functionality, affecting both environmental and host-associated microbiomes (25). In mammals, climate-sensitive dysbiosis has been shown to alter SCFA production, barrier integrity, and immune signaling—mechanisms that are increasingly recognized as modulators of seizure susceptibility (26). Such findings indicate that the gut microbiota may act as a mediator linking environmental stressors to neurological function, with temperature-dependent changes in microbial diversity reflecting adaptive or maladaptive responses depending on host resilience.

A mechanistic link between gut microbiota and epilepsy involves immune, metabolic, and oxidative pathways. Dysbiosis can activate pro-inflammatory cytokines such as IL-6, IL-1β, and TNF-α, altering neuroimmune communication and increasing neuronal excitability (19). Concurrently, reduced SCFA levels (especially butyrate) impair intestinal barrier function and microglial regulation, facilitating neuroinflammation. These effects are compounded by oxidative stress, which has been identified as a key interface between environmental insults and neuronal dysfunction in epilepsy (52) and other neurological conditions. In an Alzheimer zebrafish model, restoration of microbial balance attenuated ROS accumulation and inflammation, underscoring the reciprocal relationship between oxidative homeostasis and gut ecology (47). These convergent mechanisms strengthen the hypothesis that certain climate-induced dysbiosis could promote neuroinflammation and lower seizure thresholds.

Interestingly, not all microbiome shifts under warm conditions are deleterious. Long-term studies in environmental microbiomes demonstrate that microbial communities can maintain remarkable habitat stability, with abrupt reorganization occurring only beyond certain ecological thresholds (36). This concept of “microbial tipping points” may also apply to host-associated microbiomes, where moderate temperature or humidity fluctuations might be compensated through microbial plasticity and host adaptation, while extreme or prolonged events could precipitate dysbiosis with neurological consequences.

Despite the growing body of evidence, a major limitation is the lack of integrative mechanistic models that combine environmental, microbial, and neurological data. Most studies remain correlative and context-specific, lacking experimental validation under controlled climate conditions. Furthermore, methodological heterogeneity such as different sampling protocols, climatic metrics, and host species limits comparability. A unified framework is needed to connect macro-environmental drivers to microbial function and neurophysiological outcomes.

Clinically, understanding these interactions could have significant implications. Climate-related stressors might influence antiseizure drug pharmacokinetics, hydration status, and metabolic control. Microbiome-targeted therapies, including probiotics, dietary interventions, or fecal microbiota transplantation, may offer strategies to enhance resilience against climate-induced perturbations (51, 53). Translating concepts from environmental microbiome engineering into clinical neurology could enable the development of interventions aimed at stabilizing host microbial ecosystems under climatic stress.

Future research in this field should proceed along two complementary directions. Basic research should focus on mechanistic validation of climate-driven microbiome alterations using controlled experimental and multi-omics models, while clinical research should prioritize longitudinal human studies and interventional trials aimed at identifying climate-sensitive epilepsy phenotypes and microbiome-targeted therapeutic strategies. Basic research should therefore prioritize controlled-environment models to test climate–microbiome–brain interactions; multi-omics studies integrating microbial, immune, and neural data; longitudinal human cohorts linking climatic exposure to seizure outcomes; and microbiome-based therapeutic strategies tailored for climate-sensitive epilepsy phenotypes.

Finally, a coordinated basic-to-clinical research strategy is required to move from correlation to causation in the emerging field of climate–microbiome–epilepsy interactions. By combining mechanistic experiments, multi-omics profiling, environmental analytics, and targeted clinical interventions, future research can clarify causal pathways, identify vulnerable populations, and develop evidence-based strategies to improve neurological resilience in a warming world.
